# Sharing Data for Public Health Research by Members of an International Online Diabetes Social Network

**DOI:** 10.1371/journal.pone.0019256

**Published:** 2011-04-27

**Authors:** Elissa R. Weitzman, Ben Adida, Skyler Kelemen, Kenneth D. Mandl

**Affiliations:** 1 Children's Hospital Informatics Program at the Harvard-MIT Division of Health Sciences and Technology, Children's Hospital Boston, Boston, Massachusetts, United States of America; 2 Division of Adolescent Medicine, Children's Hospital Boston, Boston, Massachusetts, United States of America; 3 Division of Emergency Medicine, Children's Hospital Boston, Boston, Massachusetts, United States of America; 4 Department of Pediatrics, Harvard Medical School, Boston, Massachusetts, United States of America; 5 Manton Center for Orphan Disease Research, Children's Hospital Boston, Boston, Massachusetts, United States of America; Central Institute of Educational Technology, Canada

## Abstract

**Background:**

Surveillance and response to diabetes may be accelerated through engaging online diabetes social networks (SNs) in consented research. We tested the willingness of an online diabetes community to share data for public health research by providing members with a privacy-preserving social networking software application for rapid temporal-geographic surveillance of glycemic control.

**Methods and Findings:**

SN-mediated collection of cross-sectional, member-reported data from an international online diabetes SN entered into a software applicaction we made available in a “Facebook-like” environment to enable reporting, charting and optional sharing of recent hemoglobin A1c values through a geographic display. Self-enrollment by 17% (n = 1,136) of n = 6,500 active members representing 32 countries and 50 US states. Data were current with 83.1% of most recent A1c values reported obtained within the past 90 days. Sharing was high with 81.4% of users permitting data donation to the community display. 34.1% of users also displayed their A1cs on their SN profile page. Users selecting the most permissive sharing options had a lower average A1c (6.8%) than users not sharing with the community (7.1%, p = .038). 95% of users permitted re-contact. Unadjusted aggregate A1c reported by US users closely resembled aggregate 2007–2008 NHANES estimates (respectively, 6.9% and 6.9%, p = 0.85).

**Conclusions:**

Success within an early adopter community demonstrates that online SNs may comprise efficient platforms for bidirectional communication with and data acquisition from disease populations. Advancing this model for cohort and translational science and for use as a complementary surveillance approach will require understanding of inherent selection and publication (sharing) biases in the data and a technology model that supports autonomy, anonymity and privacy.

## Introduction

Diabetes is a global health threat with an evolving disease morphology whereby onset, burden and course are shifting in concert with population-wide alterations in behavioral and lifestyle factors and disease management strategies [1] [2], [3]. Intensive population-wide monitoring and longitudinal tracking of diabetes are imperative for course correcting the disease and represent a significant extension of current reporting practice and capacity.

We test a low-cost and scalable model of citizen science [4] for diabetes research and surveillance by launching and promoting a data-sharing software application into an established online international community of people with diabetes. Unprecedented uptake by consumers of online social networking (SN) through social media websites like Facebook and MySpace represents an opportunity to engage populations in citizen science health research [5]. Our attempt is to assess the willingness of members of social networks to participate, as a distributed population of citizens—lay observers and patients—in public health reporting about diabetes to augment clinical observation undertaken within structured samples. We focus on a disease-specific online SN site. This type of site provides a vehicle for communities with strong impulses to advance health and may be an important untapped resource for research: sites operate independently of formal health care and information systems, serve as virtual support groups for substantial numbers of patients and/or caregivers [6] [7] and, while not intended as channels for specific programs to influence knowledge, behavior or beliefs, nor for surveillance or research they are evolving. For example, PatientsLikeMe, a network created for patients with Amyotrophic Lateral Sclerosis [8], now actively recruits members for patient-driven observational studies and even biological sample collection.

We tested an SN-mediated approach to research sharing, focusing on a very simple idea: that we could facilitate a “data donation drive” to enable community wide surveillance of variation in a standard measure of glycemic control, the glycosylated hemoglobin (HbA1c or A1c). The A1c provides a measures of the average blood glucose present over the past three months, serving as a diagnostic marker of diabetes and is associated prospectively with risk for diabetes complications [9] [10]. Incremental reductions in A1c% have been associated with reduced risk for diabetes related complications in rigorous prospective research [11], underscoring the importance of careful tracking of this indicator. For this purpose, we developed a software application to allow members of an international online diabetes SN site to report about and share disease information for cohort and community research as part of their SN activity. We examined reactivity of the sample to outreach efforts, uptake patterns by country and state, patterns of data sharing and re-contact permissions set by users and the associations among these factors and health status—information vital to understanding selection and reporting biases for this new approach. We also undertook preliminary analyses of comparability of network-sourced aggregate data with aggregate data from a structured health reporting system and sample. We hypothesized high willingness to participate and share information and a gradient in information sharing among application users such that participants selecting highly public sharing conditions would report better diabetes health metrics than participants selecting more private sharing conditions, at least initially. We also hypothesized that despite the likely inherent selection bias suggested by the medium and model, aggregate levels of glycemic control reported by project participants would resemble aggregate metrics obtained using a national structured reporting system and sample. Our aim was to lay the groundwork for longitudinal study using an engaged cohort of social network members and test the feasibility of this model for rapid reporting of health metrics for health research and as a source of personal and contextualized feedback to the online community.

## Methods

### Test Site and Sample

The test site is www.TuDiabetes.org, an international online diabetes social network. Founded in March, 2007, it is operated by the not-for-profit Diabetes Hands Foundation. At the launch of our project, TuDiabetes had 14,678 members. The majority of website use is by members in the US (77% of website visits) but also Canada (6%), the UK (4%), Australia (1%), and other countries. In the US, California has the most visits (13%), followed by New York (8%), Texas (7%), and Florida (5%). Persons ages 18 and over are eligible for membership; younger persons are required to join with a parent/guardian. Membership comprises primarily patients with a minority of members (approximately 15%) joining as significant others or friends of persons with diabetes. The website contains news articles, blogs, and discussion forums, and allows one to create an online profile to interact with other members.

Network members are eligible to use the TuAnalyze application if they meet membership criteria for TuDiabetes–are at least 18 years old, speak and read English, have internet access and are affected by diabetes as a patient, family member or friend of someone with diabetes. The network uses a team of volunteer members to review membership applications and member activities in an attempt to limit the presence in the community of persons seeking to profit from engaging with the community in a duplicitous or non-transparent fashion.

### Software Application Overview

**Figure 1:**
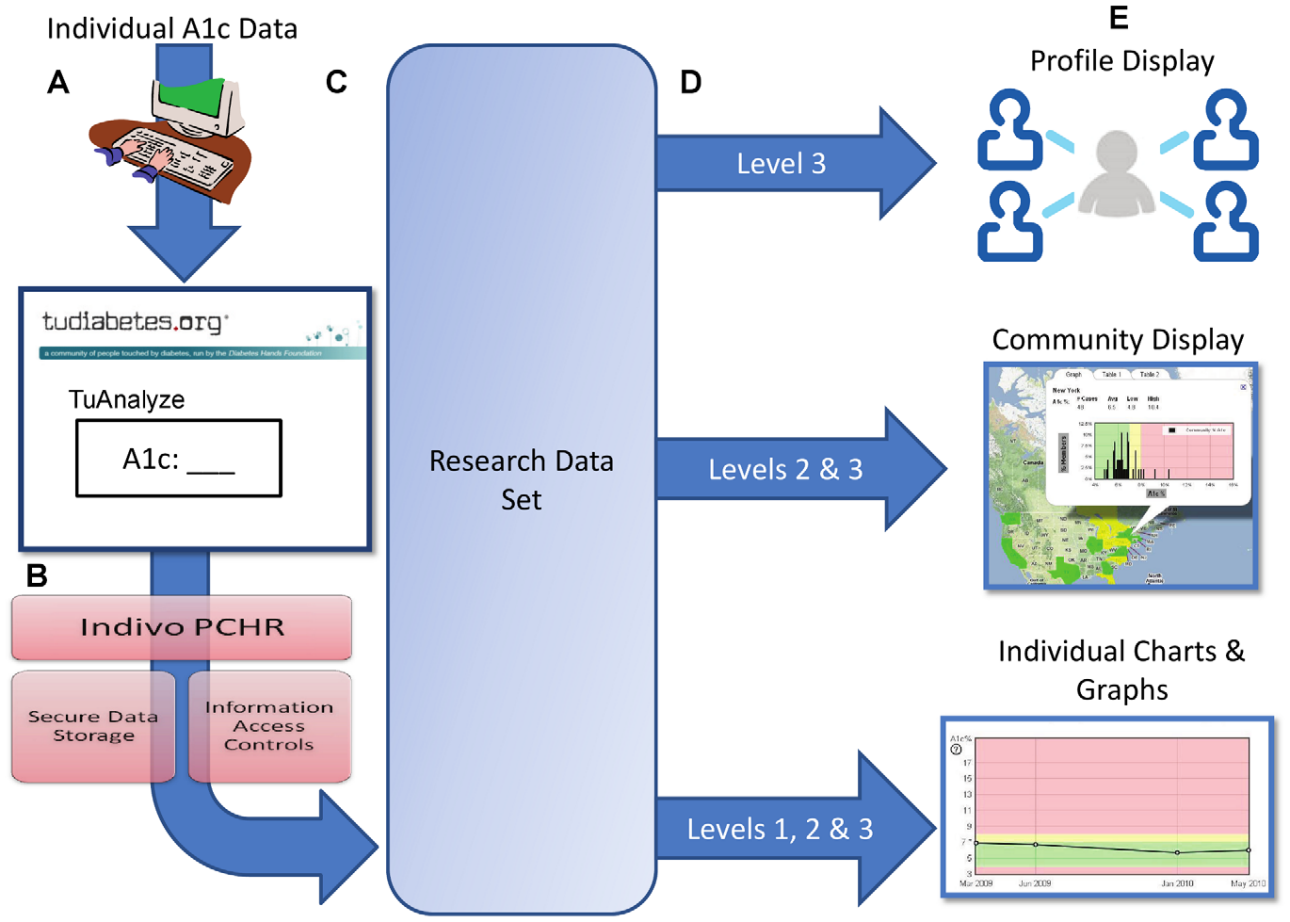
Main activity flows of the TuAnalyze application. Individuals enter A1c data into the TuAnalyze application on TuDiabetes.org, select sharing settings and consent to be re-contacted (A). Data flows into the Indivo PCHR (B) which provides secure backend, information storage and patient-controlled sharing. All data entered into the application are captured in the research data set (C); an individual's sharing setting (D) determines how and to whom their data is displayed on the TuDiabetes site (E).

The TuDiabetes network is built on Ning, a software platform for creating custom social networks. Ning implements the widely-adopted OpenSocial standard for online social networks, hence conferring generalizability to applications we develop for it. As do Facebook and MySpace members, TuDiabetes members routinely add software applications developed by third parties to their profile. We developed and deployed a novel application TuAnalyze, which enables members to report and share biomedical data with the community under a consented model. TuAnalyze is also a bidirectional communication link between the online community and a research or public health team. The application operates within the TuDiabetes website and is platform-independent. TuAnalyze uses an innovative approach to managing patient autonomy and confidentiality in that the backend of the software application is a personally controlled health record [12], [13]. We leverage the Indivo personally controlled health record's fine grained, user-managed access controls to enable consented sharing of data with the community, research teams, or public health. Figure 1 provides a schematic of the main activity flows of the application. When a user has agreed to share their information a query/poll across the personal health records returns consented results. An important feature of TuAnalyze is biosurveillance-derived display of live, aggregate, geo-referenced data back to the community for benchmarking at country, province and/or state level. Geographic areas within the map (e.g., a US state or Canadian province) illuminate with descriptive displays once a sufficient sample of participants from that area engages and shares data. Illumination of regions of the map is tied to TuAnalyze participation: a critical mass of TuDiabetes members is required to illuminate a region to protect individual identity, incent ongoing engagement, and provide a graphical and tabular data context against which engaged users can compare themselves. There is growing evidence that patients and consumers are interested in making such comparisons [8], [14]. The displays are created from aggregated permissioned data polled across personally controlled health records. They are frequently refreshed creating a near real-time “biosurveillance” display of A1c levels. Individuals sharing data can view in application graphs their A1c level plotted against the distribution in their own state.

### Information Sharing and Permission to Re-contact Users for Research

**Figure 2:**
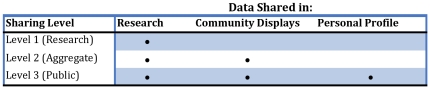
Sharing setting schematic.

Preferences for sharing information are flexible to accommodate motivators of personal and collective benefit through affording different uses and views of data. Three sharing settings are supported among which there is a hierarchical relationship (see Figure 2). As a condition of application use, all participants agree to share data for research purposes under conditions of strict identity protection (level one, the default setting). Users can also opt to share their personal information with aggregate charts, graphs and maps for display within the community where data are anonymized and individual identity is protected (level two). Finally, users can choose to share their information in the above manner and on their network profile, to be visible according to the privacy settings that govern that page (level three). In this least restrictive condition, anyone a user permits to see their profile page can see information entered in the application. Users can set their preference for being re-contacted about future research participation as part of the application interface. The default setting is to permit re-contact and prompts users to confirm or change this setting.

### Sources of Data and Health Measurement (A1c)

Study data draw on three sources and sets of measures. The first set of data is from the TuAnalyze application and includes self-reports of most recent and past A1c values, user-set selections governing research participation, privacy/sharing settings and re-contact, and TuAnalyze metadata about application use (e.g., joining date, number of A1cs entered). The main health metric, A1c, is an excellent summary statistic for personal and population monitoring of diabetes including in self-reported form [15], [16]. The application user interface supports A1c reporting on a continuous scale, with associated data fields describing date (month/year) of both A1c lab test and TuAnalyze data entry. The second data category comprises metadata from the TuDiabetes host site, specifically geography of membership for the overall community including state location for US members, obtained from an export of the member database provided by the site administrator prior to implementing the application. Geographic location is requested upon creation of an account on TuDiabetes and may be changed at any time; these data were obtained as semi-structured user-entered information and were then cleaned and integrated into the project database. The third category of information comprises the most recent National Health and Nutrition Examination Study (NHANES) 2007–2008, which includes laboratory and self-reported values for A1c among a representative adult US sample. The 1024 NHANES A1c values represent both self-report (n = 213) and lab results (n = 1003); for those individuals with both values (n = 192), the average of the two was used in comparisons. NHANES data were downloaded as a publicly available file from the Centers for Disease Control and Prevention website.

### Sharing Toward a Citizen Science Model

The approach is guided by a model for engaging a distributed population of observers in research activities through reporting, sharing and contributing labor and computing time under a collective enterprise organized around scientific discovery or gain. The design builds on the intrinsic interest within online health-related social networking sites to share information for personal or collective benefit [17], [18] and/or as an expression of “information altruism” [19] [20]. The social network mediated model is distinct from a more general sentinel surveillance model in which citizens report about their health to a central authority using a web-interface or survey, as is being done under the Gripenet project centered on influenza [21]. The TuAnalyze cycle of data entry, processing, sharing and contextualization are assumed to reinforce ongoing engagement in a virtuous cycle of collaborative research. The spirit of collective enterprise is further reinforced through:

- “branding” of the application in relation to the host social network
- promotion and reinforcing activities of the host network and restriction around use of the application and participation to SN community members
- opportunity to self-identify within the community as a participant in the project
- visualization of participants' contributed data, de-identified and aggregated, within the community pool of data
- opportunity to contextualize personal information relative to larger community aggregates.

### The Data Donation Drive

Community engagement in the effort was pursued in May 2010 through publication on the site of multiple open broadcast announcements to the community alerting them to the availability on the site of the research application and overall project. These initial promotional activities occurred during the first four weeks and included publication by the site administrator of a news article, banner text, forum posts and a blog entry about the project, and email communication to the membership. Additionally, the community newsletter featured an article about TuAnalyze. Open promotion was followed in June 2010 with more targeted outreach using Twitter to encourage continuing community uptake and emails and direct messages to location-based groups to encourage state level engagement sufficient to light up various regions of map displays. The promotional communications emphasized the voluntary nature of participation, the strict controls over privacy and sharing, and used a non-judgmental tone around sharing and levels of A1c to encourage a broad range of community members to participate.

### Analyses

The study period was the first three months of the application's availability. Analyses consider all users given the international membership of the host network and sub-analyses of US users, the largest initial user group. For US only analyses, we examined state level engagement in the application and tested for differences between the national percentage of TuDiabetes members using the application and each by-state average, testing for differences in average A1c among participants from states whose uptake patterns of the application are below, above and equivalent to the national pattern. Also for US members, we estimated the average A1c and compared that to the value estimated from the most recent NHANES study (2007–2008). We used descriptive statistics to characterize engagement with the application over time and by geography, sharing settings and diabetes health metrics. The exact binomial test was used to compare US state-level application use to the mean. ANOVA was used to compare A1c across the three groups of representative and non-representative states and the three levels of sharing. Differences in A1c by location, research contact settings, number of values entered, join period, currency of value, and source (TuAnalyze or NHANES) were examined using the two-sample t-test. Chi square tests were used to compare application settings and use by location (US and non-US). All data were analyzed using SAS version 9.2.

All study activities were reviewed and approved by the Children's Hospital Boston Committee on Clinical Investigation under a model of implied consent that was based on the pre-existing norms for sharing in the community and in alignment with the published privacy policy and terms of use of the site that clearly inform the community about conditions for sharing data and privacy protections.

## Results

### Initial uptake of the TuAnalyze application by nationality, US state

**Figure 3:**
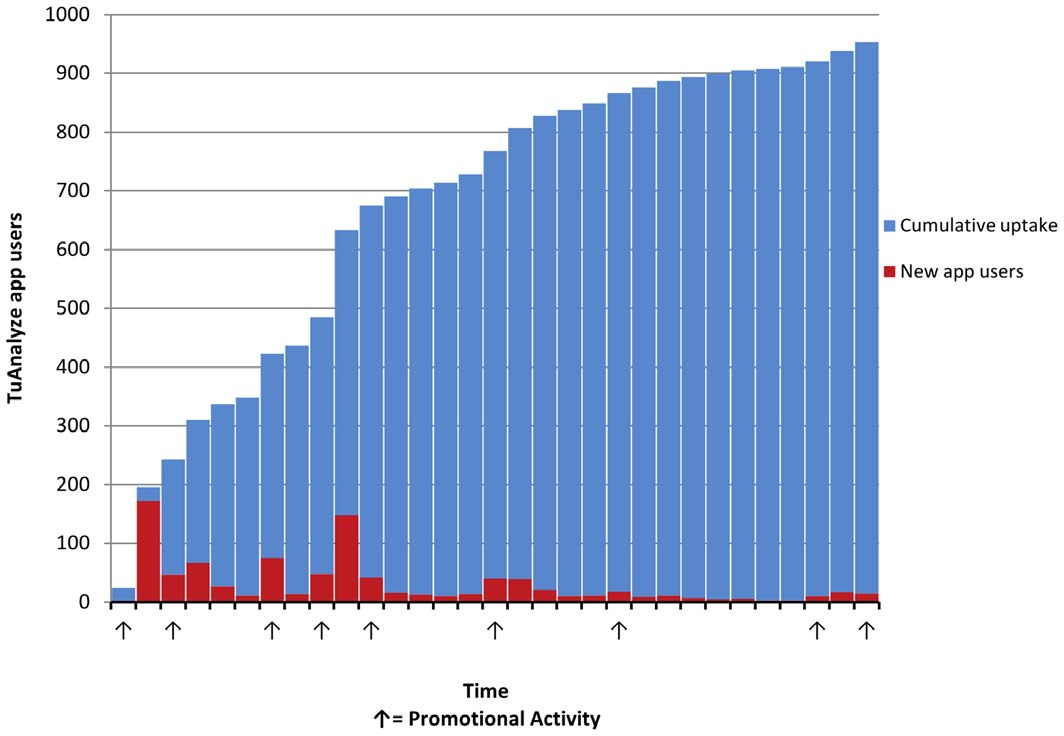
Cumulative daily uptake of the TuAnalyze application, first 31 days. *A group of users was invited to test the application in the week before its official launch; 22 of them signed up for the application during that time and these make up the “Cumulative Uptake” on Day 1.

In the initial study period, 1,136 members engaged with the TuAnalyze application and 1,062 entered into it at least one A1c value. The individuals using the application represent TuDiabetes members from 32 countries and all 50 US states. Entered values describe A1cs measured anywhere between 0 days to 7 years prior to data entry. 83.1% of the most recent values entered by users were “current”, meaning reported values were obtained within 90 days of data entry. A substantial majority of application users (89.7%) began using the application within 30 days of launch. Engagement in this initial period aligns with outreach and promotion activities to the TuDiabetes community (see Figure 3, cumulative application uptake synced to promotion in the first 30 days).

Uptake of the TuAnalyze application is on a rolling basis prospectively and estimating participation requires specification of a denominator. Of 14,678 registered TuDiabetes members at launch, 40–50% were considered to be “active” members based on application to the total site membership of the logged traffic for repeat site visits obtained using a web analytic system [22]. Thus an *unconstrained* estimate of participation is 17% (n = 1,136 of an estimated n = 6,500 active members). Alternatively, 11,019 members received a targeted newsletter promotion about the application. Thus a *constrained* estimate of initial participation is 10% (n = 1,136 of n = 11,019 targeted mail recipients). The actual participation level is likely to be somewhere between these two estimated levels.

US members make up 90% of the TuAnalyze population; Canada, Great Britain, and Australia represent the three largest populations of non-US users (respectively, 3.5%, 1%, and 0.75%). Of the US TuDiabetes population at launch (n = 10,223), approximately 4,600 were considered to be active during the initial launch period [22]. 21% (n = 959) are using TuAnalyze. The proportion of US users picking up the application at the state level was proportionate to this overall level in all but four states, one of which was underrepresented and the other three were overrepresented. Together, the membership in these four states comprised 21% of the overall TuAnalyze US users.

Multiple A1c values were entered by 14.5% of all TuAnalyze users (n = 154) upon first entering any data (n = 77) and/or in a return visit to the application (n = 90). Of all users with multiple values, 45% returned to the application to enter at least one prospectively measured A1c (i.e., a value obtained after starting to use the application). Non-US users were more likely than US users to have entered multiple measurements (OR = 1.94, 95% CI 1.2–3.2).

### Sharing and Re-contact Settings

Overall, 81.4% of TuAnalyze users chose to include their data in charts, graphs and maps describing the community with 34.1% of the total also sharing their personal A1c data on their profile page. The remaining 18.6% of users opted out of community sharing entirely but agreed to have their data included in the research data set. The distribution amongst the three privacy options (Table 1) was comparable for US and foreign users (p = 0.29). Despite the community map featuring only the United States during the study period, the proportion of users inside and outside of the US who opted to share their A1c data with the community on the aggregate map was not significantly different (p = 0.20). 95% of both the US and overall population have permitted re-contact about further research studies.

**Table 1:** Associations among glycemic control, sharing and engagement among TuAnalyze users who entered A1c data.

	N (%)		Average A1c% (SD)	
	Total	US	Total	US
	1062	959 (90.3)	6. 9 (1.3)	6.9(1.3)
**Sharing Settings**				
Public	362 (34.1)	325 (33.9)	6.8 (1.2)*	6.8 (1.2)
Aggregate	502(47.3)	460 (48)	6.9 (1.3)	6.9 (1.3)
Owner	198 (18.6)	174 (18.1)	7.1 (1.3)*	7.0 (1.2)
**Permission to Re-Contact**				
Yes	1007 (94.8)	910 (94.9)	6. 9 (1.3)	6.9 (1.2)
No	55 (5.2)	49 (5.1)	6.8 (1.4)	6.8 (1.3)
**A1c Values Entered**				
1	908 (85.5)	829 (86.4)	6.9 (1.3)**	6.9 (1.3)**
>1	154 (14.5)	130 (13.6)	6.6 (0.9)	6.6 (0.9)
**Join Period**				
First 2 Weeks	714 (67.2)	657 (68.5)	6.8 (1.2)*	6.8 (1.1)**
Later Adopters	348 (32.8)	302 (31.5)	7.0 (1.4)	7.0 (1.5)
**Dates**				
Current A1c (w/in 90 days)	886 (83.4)	797 (83.1)	6.9 (1.2)	6.9 (1.6)
Outdated A1c	176 (16.6)	162 (16.9)	6.9 (1.5)	6.9 (1.2)
**Comparability to Existing Data**				
TuAnalyze		959		6.9 (1.3)
NHANES		1024		6.9 (1.7)

*P<0.05.

**P<0.01.

### Glycemic Control

Average A1c among users was 6. 9% (Table 1) and did not differ between US and non-US TuAnalyze users (p = .69). The US states with disproportionately high or low levels of uptake did not differ from the rest of the states with respect to average A1c (p = .23). In both the total sample and the US population, users who entered multiple A1c values into the application had on average a lower most recent A1c than those who had entered only one. Very early adopters (i.e., those picking up the application within the first two weeks following its launch) also had a lower average A1c than did participants who picked up the application later in its diffusion in both groups. Among very early adopters there was no association between glycemic control and sharing/privacy setting.

In the population as a whole, less restrictive privacy settings were associated with better self-reported measures of glycemic control. Users selecting the most permissive sharing option (profile-display) had a lower average A1c (6.8%) than users with the most restrictive setting (7.1%, p = .038). However, this association was not robust (6.8% vs. 7.0% p = .058) among the US sample which may indicate insufficient power.

### Comparability

The average A1c for US TuAnalyze users (6.9%) was comparable to the unadjusted average A1c of adults with a diagnosis of diabetes or pre-diabetes reported in the 2007–2008 NHANES (6.9%, p = 0.85). There were no differences between aggregate TuAnalyze and aggregate NHANES data in subanalyses that considered NHANES modality (self-report, lab, or the average of the two).

## Discussion

It is well established that social networks transmit norms, behaviors, information and pathogens that along with other network properties, such as social support and social capital, influence health [23], [24], [25], [26]. Where reliable information about ties and health outcomes or behaviors exists, novel and important inferences about the patterning of disease in populations can be made through social network analyses [27], [28]. Where a communication channel back to a source population persists, our ambition of a rapid surveillance platform for ongoing investigation and translation of findings to action may be achieved. Toward that goal, we tested a model for engaging a distributed population of lay persons and patients in reporting about diabetes using a software application implemented for use by an online international diabetes SN community.

We found high levels of participation and sharing of personal health information for research use. There was substantial early adoption of the application with participation by 10%–17% of the overall community and 21% of the US community in the initial study period. Uptake followed targeted outreach and promotion suggesting that a rapid surveillance model run on the SN platform can stimulate engagement and suggesting also the importance of monitoring and directing promotional activity to foster diffusion and sustained engagement. Extending the model to other public health actions, such as alerting, surveying, polling around adverse events to support post-market drug safety surveillance and comparative effectiveness studies—all of which require engaged samples and a nimble structure—may be feasible. Among participants, the large majority (83%) of reported A1c measures were current when reported, further supporting use of the modality for rapid assessment of population health status. As A1c is typically measured every three months, it remains to be seen whether the data set remains current as it matures past the study period. A minority of users provided a retrospective time series of their A1c when they first used the application. The low prevalence of this was not surprising given the application user interface instructed users to enter their “most recent A1c” albeit the application supports entry of a time series of measures. The values for the most recent A1c reported by users who provided a time series were lower on average than those reported by their peers entering only one value. Compared to users reporting one value, users entering a time series may be more vigilant, better organized, or have better access to health information and testing resources. Though differences in A1c between groups seem small, on a population level these differences translate to substantial health impacts.

Four fifths (83.1%) of users are actively choosing to share their data with others in the community and switched from the default sharing setting (research only) to either sharing data with community charts or graphs or profile display. Thus, a strong norm of sharing for research does not equal a blanket norm of openness. This is consistent with prior findings of high willingness to share personal health information for research conditioned by perceptions of autonomy, anonymity, context and purpose [29]. Provision for user control over data sharing through the application is a marked improvement over standard practice among diabetes social networking sites wherein sites commonly share member data and provide few if any user controls [6]. As hypothesized, users selecting the most restrictive sharing settings had on average worse self-reported measures of glycemic control than users who selected more public/less restrictive settings. Thus, data shared within the community may be slightly skewed toward a better overall health metric. This may reflect myriad factors including embarrassment, inhibition, or concern for adverse consequences from disclosure, as found in prior research on consumer-centered health information technologies [14]. Ability to discern these biases is vital to successful use of this approach and will help protect against validity threats to inferences made about data from the community. Equally vital is learning how best to engage populations in worse health that may not yet be participating and that may require a greater understanding of the approach and its privacy-preserving provisions.

In the US, differences in engagement with the application by geography were few and not associated with health status. While the majority of users are from the US, the model has captured an international community and engagement may grow once country or regional participation is sufficient to trigger international illumination of mapping displays which provide incentive and context.

Unadjusted aggregates of glycemic control in the community were not different from unadjusted aggregates from the most recent NHANES panel and this finding holds true for lab and self-reported subsamples of NHANES data. Before inferences about comparability are drawn, additional data that describe disease type, course, history and demographics will need to be collected and controlled in analyses. This will be possible as the application matures to include a survey tool and other features. For this first report, we note that the TuAnalyze model engaged an equivalently sized sample in a highly accelerated time period using a community-based approach that supports permissioned recontact. This model enriched by additional demographic information may comprise a rapid assessment complement to traditionally structured research efforts.

The TuAnalyze software will allow integration of professionally-sourced health system data in the near future through activation of the personally controlled health record system platform on the backend. This expansion, plus the integration of survey data into the application interface, will afford opportunity to validate patient entered information against health services data and enable a truly comprehensive diabetes monitoring system with twinned lenses of clinic and citizen-consumer.

### Limitations

Promising early results should be viewed in the context of limitations. The TuAnalyze population comprises consumers who may be highly motivated to achieve good glycemic control and whose degree of technological savvy and digital comfort may be unrepresentative of the general population of persons with diabetes. Participation biases limit our ability to generalize but they enable this research and learning about them is vital to advancing this model. Data on participation in a distributed community with changing membership and participation are necessarily limited by challenges in ascertaining a reliable denominator that describes persons exposed to the site or active during a given time period. Levels provided for this analysis are bracketed by likely upper and lower bounds but are estimates: reliable counts of registered members are available but not all members were active during the study period or “at risk” of being notified about a new application. Data are self-reported and analyses do not yet adjust for demographics, diabetes type and disease course factors. Collection of these data was not supported under the initial application which prioritized a test of engagement with the model and near real-time processing and return of A1c in a model that supports contextualized reporting back to the community. Future work will include a broader set of analyses on demographically adjusted data, enabled by expanding the TuAnalyze application to include a survey tool and other features. The test site defines itself as a single community and we need to study whether the findings generalize to other online communities, including ones that may contain patients with other health problems including diseases that may be highly socially stigmatized. Nevertheless, the site and sample represent considerable international and geographic spread.

### Conclusions

The participatory model employed in TuAnalyze is centered on building a research *relationship* with an engaged cohort to foster prospective study, ongoing communication and flexibility sufficient to accommodate an evolving study protocol or measurement model and diffusion and translation of findings. Our approach, wherein data is sourced from a volunteer, consented cohort, is distinct from data mining of web content where users have no voice, control or opportunity for follow-up. It also differs from registries of clinically tethered populations which provide few opportunities for real-time patient input or expansion beyond institutionally bound patient populations. Clinical registries provide vital clinically observed metrics but they typically lack patient-reported outcomes and generally do not provide registry data back to patients; moreover, the timing and tempo of clinical interviews and observations follow a research visit timeline as opposed to the calendar of the patient and her experiences. The TuAnalyze model, which provides for patient reporting and aggregate feedback on a flexible time schedule could provide a boon to public health and research and complement these other approaches.

Leveraging the medium's power to sustain engagement and foster consented reporting and communication may offset cost, labor and processing demands associated with large sample prospective data collection and ameliorate research challenges related to: a) recruiting and maintaining study cohorts and samples; b) moving beyond inflexible and single disease data models that are difficult to modify or extend once in the field; c) responding rapidly and at scale to emerging health phenomena or findings; and, d) “closing the loop” between collection and analysis of research data and translation or communication of findings to source populations. No one model may be sufficient to address the problem of diabetes but strategic use of a range of approaches may help “steer the ship” as we address a global pandemic.
